# Cyr61 is involved in neutrophil infiltration in joints by inducing IL-8 production by fibroblast-like synoviocytes in rheumatoid arthritis

**DOI:** 10.1186/ar4377

**Published:** 2013-11-13

**Authors:** Xianjin Zhu, Lianbo Xiao, Rongfen Huo, Jie Zhang, Jinpiao Lin, Jun Xie, Songtao Sun, Yong He, Yue Sun, Zhou Zhou, Baihua Shen, Ningli Li

**Affiliations:** 1 Shanghai Institute of Immunology, Institute of Medical sciences, Shanghai Jiao Tong University School of Medicine, 280 South Chongqing Road, Shanghai 200025, P. R. China; 2 Shanghai Guanghua Rheumatology Hospital, 540 Xinhua Road, Shanghai 200025, P. R. China; 3 Affiliated Union Hospital of Fujian Medical University, 29 Xinquan Road, Fuzhou 350001, P. R. China

## Abstract

**Introduction:**

It is well known that neutrophils play very important roles in the development of rheumatoid arthritis (RA) and interleukin (IL)-8 is a critical chemokine in promoting neutrophil migration. We previously showed that increased production of Cyr61 by fibroblast-like synoviocytes (FLS) in RA promotes FLS proliferation and Th17 cell differentiation, thus Cyr61 is a pro-inflammatory factor in RA pathogenesis. In this study, we explored the role of Cyr61 in neutrophil migration to the joints of RA patients.

**Methods:**

RA FLS were treated with Cyr61 and IL-8 expression was analyzed by real-time PCR and ELISA. The migration of neutrophils recruited by the culture supernatants was determined by the use of a chemotaxis assay. Mice with collagen-induced arthritis (CIA) were treated with anti-Cyr61 monoclonal antibodies (mAb), or IgG1 as a control. Arthritis severity was determined by visual examination of the paws and joint destruction was determined by hematoxylin-eosin (H&E) staining. Signal transduction pathways in Cyr61-induced IL-8 production were investigated by real-time PCR, western blotting, confocal microscopy, luciferase reporter assay or chromatin immunoprecipitation (ChIP) assay.

**Results:**

We found that Cyr61 induced IL-8 production by RA FLS in an IL-1β and TNF-α independent pathway. Moreover, we identified that Cyr61-induced IL-8-mediated neutrophil migration *in vitro*. Using a CIA animal model, we found that treatment with anti-Cyr61 mAb led to a reduction in MIP-2 (a counterpart of human IL-8) expression and decrease in neutrophil infiltration, which is consistent with an attenuation of inflammation *in vivo*. Mechanistically, we showed that Cyr61 induced IL-8 production in FLS via AKT, JNK and ERK1/2-dependent AP-1, C/EBPβ and NF-κB signaling pathways.

**Conclusions:**

Our results here reveal a novel role of Cyr61 in the pathogenesis of RA. It promotes neutrophil infiltration via up-regulation of IL-8 production in FLS. Taken together with our previous work, this study provides further evidence that Cyr61 plays a key role in the vicious cycle formed by the interaction between infiltrating neutrophils, proliferated FLS and activated Th17 cells in the development of RA.

## Introduction

Human rheumatoid arthritis (RA) is a systemic inflammatory disease that involves hyperplasia of synovial tissues (ST) and structural damage to cartilage, bone and ligaments [1]. Although the etiology and pathogenesis of RA are still unclear, there are many inflammatory cells accumulated in the synovial fluid (SF) and involved in the pathogenesis of RA [2]. It is known that neutrophils are the most abundant cells present either in the SF of the affected joints or at the pannus/cartilage interface [3,4]. Studies have shown that infiltrating neutrophils contribute to autoimmune arthritis development and severity [5]. In animal models, neutrophil depletion by anti-Gr1 antibodies, an antibody for mouse neutrophils, can protect mice from arthritis; furthermore, injection of anti-Gr1 antibodies into mice after disease onset can impair the progression of arthritis [3,6]. Moreover, blocking neutrophil development, for example, genetic deficiency of G-CSF or the G-CSF receptor, which are both critical for neutrophil development, can protect mice from collagen-induced arthritis (CIA) [7,8]. Treatment with anti-leukoproteinase (a physiologic inhibitor of neutrophil serine proteases) not only reduces arthritis incidence and inflammation but also has a protective effect against cartilage and bone erosion [9,10]. In RA, neutrophils are commonly recruited into diseased joints by chemoattractants and enhance tissue damage [2,4,5]. Notably, recent studies have shown that neutrophils can release IL-17 in inflamed ST [11,12]. Together, these results suggest that neutrophils play important roles in the pathogenesis of RA and that affecting neutrophil migration to the diseased joints can decrease severity of RA.

IL-8/CXCL8, a potent 8.5-kDa chemoattractant for neutrophils, plays a pivotal role in the recruitment and activation of neutrophils and is considered to be the most important inflammatory chemokine associated with arthritis [13,14]. There is a positive correlation between IL-8 and the number of neutrophils in the SF of RA patients [15,16]. Consistently, it is found that IL-8 or its counterparts in animals is essential for inflammation mediated by neutrophils [17,18]. For example, MIP-2 (a counterpart of human IL-8) is increased in the hind paws of CIA mice and correlates with the number of accumulated neutrophils, and administration of MIP-2 antibody (Ab) weakens inflammation of hind paws [18]. These results indicate that IL-8 and its relative chemokines are directly involved in the pathogenesis of RA. IL-8 production is induced by many inflammatory cytokines in RA fibroblast-like synoviocytes (FLS), such as IL-1β [19], TNF-α [20,21] and IL-17 [21], but whether there are other IL-8 expression inducers remains unknown.

Cyr61/CCN1 is a product of an immediate early gene and functions in mediating cell adhesion and inducing cell migration [22-24]. As a secreted extracellular matrix (ECM) protein, Cyr61 has much potential for activation via interacting with distinct integrins in different cells [25-27]. We reported previously that the expression of Cyr61 is greatly enhanced in FLS from RA patients, and this increased expression of Cyr61 in turn acts to further stimulate FLS proliferation and induces Th17 differentiation by promoting IL-6 production in RA [28,29]. However, whether Cyr61 has any effect on IL-8 production and plays any roles in inflammation mediated by infiltrating neutrophils in RA has not yet been explored.

In this study, we found that Cyr61 stimulated IL-8 production by FLS in an IL-1β and TNF-α independent pathway. Cyr61 has the ability to enhance the binding of AP-1, C/EBPβ and NF-κB to the IL-8 promoter via an AKT, JNK and ERK1/2 dependent signaling pathway. Moreover, we determined that Cyr61-induced IL-8 mediated neutrophil migration *in vitro*. Using a CIA animal model, we found that blocking Cyr61 action with a monoclonal antibody (mAb, 093G9) reduced MIP-2 production, decreased neutrophil migration, and remarkably ameliorated disease progression in CIA mice. In conclusion, Cyr61 plays a critical role in stimulating IL-8 production by FLS in RA and contributes to recruitment of neutrophils. As IL-8 is commonly induced by IL-1β and TNF-α in the development of RA, our results indicate that Cyr61 is a novel IL-8 production inducer. Taken together with our previous work, this report provides new evidence that Cyr61 participates in RA pathogenesis as a pro-inflammatory factor and plays a key role in the vicious cycle formed by cross-talk among activated Th17, proliferated FLS and infiltrating neutrophils in the development of RA.

## Methods

### Animals

Male, DBA/1 J mice, six- to eight-weeks old, were purchased from the Shanghai Laboratory Animal Center, Chinese Academy of Science. Mice were maintained under pathogen-free conditions. All experiments were performed in accordance with guidelines and approved by the Animal Care and Use Committee of Shanghai Jiaotong University School of Medicine (2013028).

### Patients and specimens

A total of 46 RA patients (6 men and 40 women, 30- to 84-years old, mean and SD 57 ± 12 years) were included in the study. The disease duration of the RA patients was 16 ± 9 years. The diagnosis of RA was based on the revised criteria of the American College of Rheumatology [30]. The control subjects were 27 osteoarthritis (OA) patients fulfilling the diagnostic criteria of OA proposed by Altman [31]. Clinical characteristics of RA and OA patients are given in Additional file 1: Table S1. ST were obtained from patients and FLS were cultured and identified as reported previously [28]. SF and cell culture supernatants were collected as reported previously [28]. The study was approved by the Institutional Medical Ethics Review Board of the Shanghai Jiaotong University School of Medicine and informed consent was obtained from each of the individuals.

### Synovial fluid and synovial tissue cell preparation and flow cytometric analysis

To prepare single-cell suspensions from SF and ST, SF specimens were centrifuged at 500 g for 10 minutes, and cells were collected, counted and resuspended in phosphate-buffered saline (PBS) for flow cytometric analysis; ST specimens were minced into small pieces and incubated for two hours with 1 mg/ml type I collagenase (Sigma-Aldrich, Bornem, Belgium) in (D)MEM at 37°C, then cells were collected by filtering the suspension through nylon mesh and immediately used for flow cytometric analysis. For surface markers staining, fluorescence conjugated CD3, CD11b, CD14, CD15, CD16 and CD19 (eBiosciences, San Diego, CA, USA) antibodies were used. Flow cytometry was performed using a FACS Calibur cytometer (BD Biosciences, San Jose, CA, USA) and analyzed using Cellquest software (BD Biosciences).

### Real-time PCR analysis

Total RNA was extracted from cells and real-time PCR was performed as previously reported [28]. Briefly, total RNA was extracted from specimens using a Tripure isolation reagent (Roche Diagnostics, Indianapolis, IN, USA), according to the manufacturer’s instructions. The RNA quality and quantity were evaluated by a NanoDrop ND-1000 Spectrophotometer (NanoDrop, Wilmington, DE, USA). The integrity of RNA was appraised with gel analysis for the intact 28S and 18S ribosomal RNA. Messenger RNA (mRNA) was converted to cDNA using a RevertAidTM First Strand cDNA Synthesis Kit (Thermo Scientific, Glen Burnie, MD, USA) according to the manufacturer’s instructions. Real-time PCR was performed using SYBR Green Master Mix (Applied Biosystems, Foster City, CA, USA) according to the manufacturer’s instructions. The primers used in this study are shown in Additional file 1: Table S2.

### RNAi knockdown of gene expression

Cyr61, IL-1β and TNF-α small interfering RNA (siRNA, Additional file 1: Table S3) were designed and synthesized at Shanghai GenePharma (Shanghai, China) and gene knockdowns were performed as previously reported [28,29]. In brief, FLS were cultured in 24-well plates. A transfection mixture of siRNA oligonucleotides and Lipofectamine 2000 reagent (Invitrogen, Carlsbad, CA, USA) in serum-free medium was added to medium-aspirated cells for four hours. Then, the medium was replaced with complete (D)MEM containing 10% fetal bovine serum for an additional 24 hour incubation.

### Probing of signaling pathways involved in Cyr61 induced IL-8 production

Special inhibitors of the NF-κB and mitogen-activated protein kinase (MAPK) signaling pathways were purchased from Sigma-Aldrich and used to analyze Cyr6-induced IL-8 production. Briefly, 4 μM pyrrolidine dithiocarbamate (PDTC; an inhibitor of NF-κB activation), 10 μM SB203580 (an inhibitor of p38 MAPK), 1 μM PD98059 (an inhibitor of ERK1/2), or 20 μM SP600125 (an inhibitor of JNK) was added to the cell culture together with 5 μg/ml Cyr61 at the same time; then expression of IL-8 was determined using real-time PCR and the concentration of IL-8 in the supernatant was evaluated by ELISA. The activations of AKT, JNK, ERK1/2 and NF-κB were analyzed using western blotting with specific antibodies.

### ELISA

The concentration of IL-8 in the cell culture supernatant and SF was determined by a sandwich ELISA (R&D Systems, Minneapolis, MN, USA) according to the manufacturer’s instructions. The level of Cyr61 was measured by ELISA as described previously [28].

### Western blot analysis

Protein immune blotting was performed as described previously [28]. In brief, tissue or cell lysates were separated by SDS–PAGE electrophoresis and then transferred to polyvinylidene fluoride (PVDF) membranes (Millipore Corporation, Bedford, MA, USA) at 100 v for 90 minutes. The phosphorylation of AKT, JNK, ERK1/2 and NF-κB and the expression of MIP-2 were analyzed using specific antibodies (Cell Signaling Technology Inc, Beverly, MA, USA). After washing with PBS, the membranes were incubated with horseradish peroxidase (HRP)-conjugated goat anti-rabbit immunoglobulin G (IgG) at room temperature for one hour followed by washing with PBS. The target proteins were examined with an ECL system (Millipore Corporation, Bedford, MA, USA) and visualized with autoradiography film.

### Confocal laser scanning fluorescence microscopy assay

NF-κB nuclear translocation in FLS was studied with a confocal laser scanning fluorescence microscopy (LSM510; Zeiss, Jena, Germany) technique as described before [29]. In brief, FLS grown on glass coverslips were stimulated with 5 ug/ml Cyr61 for 30 minutes and fixed with acetone. The fixed cells were stained overnight with anti-NF-κB p65 antibody (Cell Signaling Technology Inc) and incubated for one additional hour with a PE-labeled secondary antibody (Santa Cruz Biotechnology, Santa Cruz, CA, USA). After washing, cells were incubated for three minutes with 0.25 mg/ml of 4,6-diamidino-2-phenylindole and examined using an LSM 510 confocal fluorescence microscope.

### Neutrophil isolation

Neutrophils were isolated from peripheral blood of healthy donors according to the manufacturer’s instructions. In brief, venous blood was drawn and neutrophils were isolated immediately by Polymorphprep (Axis-Shield PoC AS, Oslo, Norway). After lysis of the erythrocytes, the neutrophils were harvested, washed twice with physiological saline and resuspended in RPMI 1640 medium supplemented with 10% fetal bovine serum at a cell concentration of 10^6^/ml. The purified cells consisted of a more than 95% pure population of viable neutrophils, as assessed by morphology and the trypan blue exclusion test.

### Chemotaxis

Chemotaxis was assessed using 24-transwell Boyden chambers of 3 μm pore size (Corning Costar, Cambridge, MA, USA) for neutrophils. FLS were plated in 24-well plates and stimulated with 5 μg/ml Cyr61 (PeproTech, Rocky Hill, NJ, USA) for 48 hours. Then, the culture supernatant was harvested and pre-incubated with anti-IL-8 mAb, (5 μg/mL, PeproTech) or control mAb (5 μg/ml, PeproTech) for one hour. Then, the treated supernatant was added to the lower chambers, while neutrophils were added to the top chambers for incubation for another 90 minutes at 37°C in a humidified atmosphere with 5% carbon dioxide. The filters were fixed with ethanol and stained with crystal violet. The chemotactic response was then determined by evaluating the number of cells that had migrated through the entire thickness of the filter. Triplicate chambers were used in each experiment and five fields were examined in each filter. The results were expressed as the chemotactic index, being the number of cells that migrated towards the sample divided by the number of cells that migrated towards the control medium.

### Construction of luciferase reporter plasmids

The 181 bp IL-8 promoter sequences (-135 to +46) were PCR amplified from human genomic DNA using the following primers, IL-8 (WT)-Forward: 5′-GTGAGATCTG AAGTGTGATGACTCAGG-3′, which contains an artificial BglII site, and IL-8 (WT)-Reverse: 5′-GTGAAGCTTGAAGCTTGTGTGCTCTGC-3′, which contains an artificial HindIII site [32]. The PCR product was then digested with BglII/HindIII and inserted into the corresponding restriction sites of the luciferase reporter plasmid pGL3-Basic (Promega, Fitchburg, WI, USA) to generate IL-8 (WT) Luc. To generate the IL-8 (mAP-1) Luc, IL-8 (mNF-κB) Luc and IL-8 (mC/EBP) Luc vector that contains the same IL-8 promoter sequences but with mutation that distorts the AP-1, NF-κB and C/EBP consensus, the forward primers (Additional file 1: Table S4) were used together with IL-8 (WT)-Reverse [33]. The PCR products were again digested with BglII/HindIII and ligated into pGL3-Basic.

### Cell culture, transfection and reporter assay

Human skin fibroblasts (HSFs) were cultured in (D)MEM supplemented with 10% fetal bovine serum. For transient transfections, cells were grown to 70% to 80% confluence in 24-well dishes and maintained serum-free prior to transfection; then, cells were transfected with IL-8WT, IL-8mAP-1, IL-8mC/EBP or IL-8mNF-κB along with pRL-TK using the liposome–mediated method with Lipofectamine 2000 reagent (Invitrogen) according to the manufacturer’s instructions. After a 24-hour incubation period, cells were treated with Cyr61 (5 μg/mL) for an additional two hours, at which time luciferase activity was measured using a Dual-Luciferase Reporter Assay System (Promega) according to the manufacturer’s instructions.

### Chromatin immunoprecipitation assay

For chromatin immunoprecipitation (ChIP) assay, FLS cells, either with or without Cyr61 protein (5 μg/mL) stimulation, were cross-linked by formaldehyde fixation. Following cellular and nuclear lysis, isolated chromatin was sheared by sonication and subsequently incubated overnight at 4°C with antibodies against c-Jun, NF-κB p65 (Cell Signaling Technology Inc, Danvers, MA, USA), C/EBPβ (Santa Cruz Biotechnology), or control rabbit IgG (PeproTech). Immunocomplexes were subjected to cross-link reversal, extracted and precipitated as described in the protocol according to the manufacturer’s instructions. The eluted DNA and the aliquots of chromatin prior to immunoprecipitation (input) were subjected to semiquantitative PCR. The PCR primers were used for amplifying IL-8 promoters (-125 to +11) with the following sequences: -125 forward, 5′-ACTCAGGTTTGCCC TGAGGGGA-3′ and +11 reverse, 5′-TGCCTTATGGAGTGCTCCGGTG -3′. The PCR conditions were as follows: one cycle at 95°C for five minutes; 34 cycles at 95°C for 30 seconds, 65°C for 30 seconds, and 72°C for one minute; one cycle at 72°C for five minutes. PCR products were separated by 2% agarose gel containing ethidium bromide. Densitometry was used to quantify the PCR results, and all results were normalized by respective input values.

### Establishment and treatment of collagen-induced arthritis

CIA was induced as described previously [34]. Briefly, male DBA/1 J mice were injected intradermally with 150 μg of chicken type II collagen (Chondrex, Redmond, WA, USA) in 0.05 M acetic acid emulsified in Freund’s complete adjuvant. Booster injections were administered on day 21 with a total of 75 μg collagen II in Freund’s incomplete adjuvant. Joint inflammation was evaluated on a scale of 1 to 4 [35], with a maximum clinical score of 16 per mouse. Mice were treated with control IgG1 or anti-Cyr61 mAb 093G9 generated in our laboratory (200 μg/mouse) twice a week when the score reached 2.

### Hematoxylin-eosin staining

The joints were removed from sacrificed CIA mice and fixed in 10% phosphate-buffered formalin, decalcified in 10% ethylenediaminetetraacetic acid (EDTA), embedded in paraffin, stained with H & E and examined by light microscopy according to standard protocols.

### Immunohistochemistry

Slides were deparaffinized through a series of xylene baths and rehydrated through graded alcohols. The sections were then immersed in methanol containing 0.3% hydrogen peroxide for 20 minutes to block endogenous peroxidase activity and incubated in 2.5% blocking serum to reduce nonspecific binding. Sections were incubated overnight at 4°C with anti-human CD15 mAb or anti-mouse Gr-1 mAb (eBiosciences); mouse IgM or rat IgG was used as negative control in the study. Slides were then incubated in anti-mouse IgM HRP or anti-rat IgG HRP. Vector NovaRED substrate (Vector Labs, Burlinghame, CA, USA) was used as the peroxidase substrate and slides were counter-stained with a hematoxylin solution. Stained sections were dehydrated and then mounted by light microscopy.

### Statistical analysis

All experiments were performed in triplicate. The difference among groups was determined by analysis of variance (ANOVA) and comparison between two groups was analyzed by the *t*-test using the GraphPad Prism 4.0 (GraphPad Software, In c., San Diego, CA, USA). A value of *P* <0.05 was considered statistically significant.

## Results

### Neutrophils were abundant in inflamed joints of patients with RA

**Figure 1:**
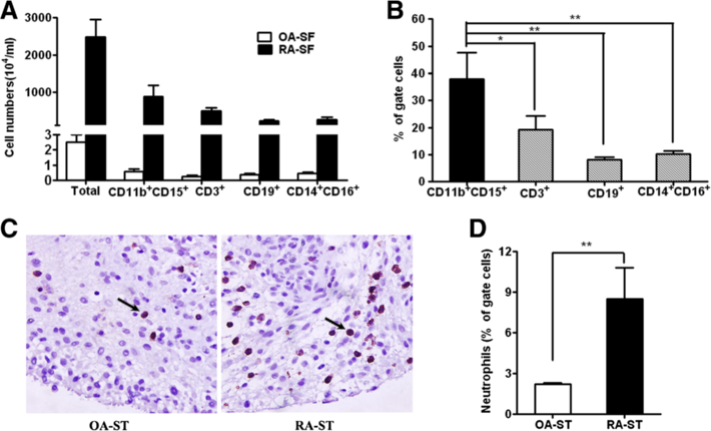
**Neutrophils are abundant in inflamed joints of patients with RA.** The amount **(A)** and percentage **(B)** of neutrophils (CD11b^+^CD15^+^) in synovial fluid (SF) from RA and osteoarthritis (OA) patients detected by FCS. **(C)** Representative photomicrographs showing immunohistochemical staining of OA and RA synovial tissues with neutrophil marker anti-CD15 Ab. Arrow points to representative neutrophils (red). Original magnification × 400. **(D)** Neutrophil infiltration in joint tissue sections detected by FCS. Isolation and staining of cells from ST as described in Methods. Data are representative of at least three independent experiments. * P <0.05, ** P <0.01. Ab, antibody; FCS, flow cytometry sorting; RA, rheumatoid arthritis.

As large numbers of studies have identified a variety of different cells involved in the pathogenesis of RA [2], we first investigated the profile of infiltrating inflammatory cells in SF from RA and OA patients. The results showed that there were large numbers of leukocytes, including a population of CD11b^+^CD15^+^, CD3^+^, CD19^+^ and CD14^+^CD16^+^ cells in RA SF. In contrast, few to no cells were detectable in SF from OA patients (Figure 1A). Further analysis showed that 25% to 65% of the examined cells were CD11b^+^CD15^+^ neutrophils, which were significantly higher than other infiltrating cells (Figure 1B), suggesting that neutrophils might be the dominant type of infiltrating inflammatory cells in SF of RA patients. Next, we examined the occurrence and distribution of neutrophils in ST of RA patients. The results showed there were large numbers of CD15^+^ neutrophils in RA ST (Figure 1C). To further identify the population of infiltrating cells, we employed flow cytometry analysis and the results revealed remarkable leukocyte infiltration, including the population of CD11b^+^CD15^+^, CD3^+^, CD19^+^ and CD14^+^CD16^+^ cells in RA ST (Figure 1D and Additional file 1: Figure S1). CD11b^+^CD15^+^neutrophils were the dominant infiltrating inflammatory cells in ST of RA patients. These results demonstrate that inflamed joints of RA patients are heavily infiltrated with neutrophils, which is consistent with previous reports [36].

### Cyr61 induced IL-8 production by FLS of RA patients

**Figure 2:**
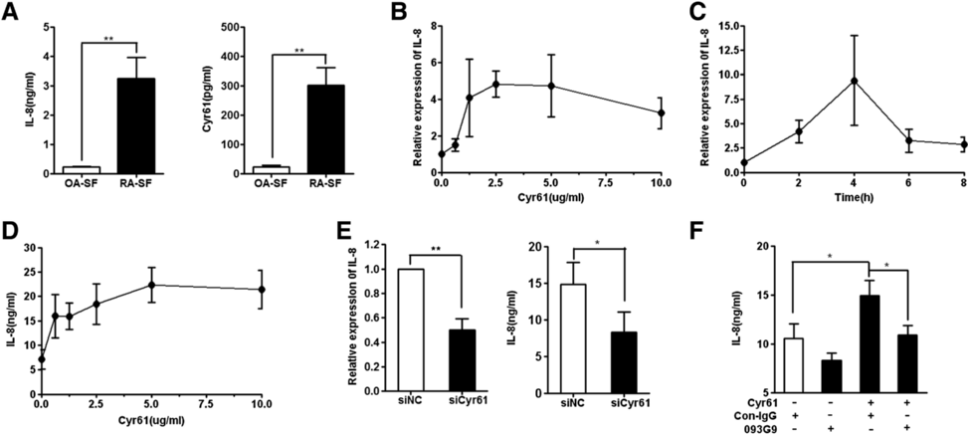
**Cyr61 promotes IL-8 production in RA FLS. (A)** IL-8 and Cyr61 levels in SF from RA patients (n = 21) and OA patients (n = 11) were detected by ELISA. **(B)** IL-8 mRNA expression in RA FLS stimulated by Cyr61 (0.62, 1.25, 2.5, 5, 10 μg/ml) for two hours was determined by real-time PCR using a housekeeping gene as an endogenous control. **(C)** IL-8 mRNA expression in RA FLS stimulated by 5 μg/ml Cyr61 (0, 2, 4, 6, 8 hours) was determined by real-time PCR. **(D)** The concentration of IL-8 in supernatant was evaluated by ELISA. FLS were seeded into 24-well plates and stimulated with exogenous Cyr61 (0.62, 1.25, 2.5, 5, 10 μg/ml) for 48 hours. **(E)** The expression of IL-8 mRNA and protein in Cyr61-knockdown FLS was detected by real-time PCR and ELISA, respectively. FLS were transfected with siCyr61 or siNC as described in Methods. After siRNA transfection, FLS were stimulated with 5 μg/ml Cyr61 for 2 hours and analyzed for IL-8 expression by real-time PCR and supernatants from FLS stimulated with 5 μg/ml Cyr61 for 48 hours were analyzed for IL-8 production by ELISA. **(F)** Cyr61-stimulated production of IL-8 by FLS was inhibited by 093G9. FLS were pretreated with 20 μg/ml anti-Cyr61 monoclonal antibody (named 093G9) or control IgG for one hour. After pretreatment, FLS were stimulated with 5 μg/ml Cyr61 for 48 hours and supernatants were analyzed for IL-8 production by ELISA. Data represent the mean ± SEM of at least three independent experiments. * P <0.05, ** P <0.01. FLS, fibroblast-like synoviocytes; OA, osteoarthritis; RA, rheumatoid arthritis; SEM, standard error of the mean; siNC, control siRNA.

IL-8 is one of the most neutrophil chemoattractant molecules and plays a very important role in pathogenesis mediated by neutrophils in RA [5,37]. A number of cells are known to produce IL-8, including macrophages and fibroblasts [20]. Given that we have previously shown that Cyr61 induces IL-6 production in FLS, which further drive Th17 differentiation and enhance inflammation of RA [28,29], we further explored whether Cyr61 may also stimulate IL-8 production by FLS. We set up an *in vitro* cell culture system using FLS isolated from RA patients and analyzed the levels of IL-8 and Cyr61 in SF obtained from RA and OA patients. The results showed that the levels of IL-8 and Cyr61 were higher in RA SF than in OA SF (Figure 2A), consistent with other reports [15,16,28]. After confirming that RA SF contained higher levels of IL-8 and Cyr61, we next tested the potential effect of Cyr61 on the expression of IL-8 by FLS of RA patients. The results showed that Cyr61 significantly stimulated IL-8 mRNA expression in FLS (Figure 2B and 2C). Consistent with these observations, ELISA showed that the concentration of IL-8 in FLS culture supernatant was significantly increased upon addition of exogenous Cyr61 (Figure 2D). To further examine the autocrine role of Cyr61 in the regulation of IL-8 expression by FLS, we performed specific siRNA to knockdown Cyr61 expression in FLS [28]. The results showed that IL-8 mRNA expression was remarkably reduced in Cyr61-knockdown FLS. The reduction of IL-8 production by FLS upon Cyr61 knockdown was also confirmed by ELISA measurement of IL-8 protein levels in culture supernatant (Figure 2E). Next, we treated FLS with an anti-Cyr61 mAb (named 093G9) and results showed that 093G9 could block the effect of Cyr61 on IL-8 production by FLS (Figure 2F). These data indicate that Cyr61 can induce IL-8 expression by FLS of RA patients, which may partly contribute to the higher concentration of IL-8 observed in RA SF.

### IL-1β and TNF-α were not involved in Cyr61-induced IL-8 production by FLS

**Figure 3:**
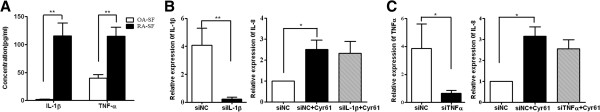
**IL-1β and TNF-α are not involved in Cyr61-induced IL-8 expression in FLS. (A)** The protein levels of IL-1β and TNF-α in SF from RA and OA patients were detected by ELISA. **(B)** IL-8 mRNA expression in IL-1β-knockdown FLS was detected by real-time PCR. Left panel, the sequence for siIL-1β (small interfering RNA against IL-1β) was evaluated. The IL-1β mRNA expression in FLS transfected with siIL-1β (black bar) or siNC (control, open bar) was measured by real-time PCR. Right panel, FLS were transfected with siIL-1β or siNC for 4 hours, cultured for 20 hours and incubated for an additional 2 hours in the presence or absence of Cyr61 (5 μg/ml). IL-8 mRNA expression in FLS was detected by real-time PCR. **(C)** IL-8 mRNA expression in TNF-α-knockdown FLS was detected by real-time PCR. Data represent the mean ± SEM of at least three independent experiments. * P <0.05, ** P <0.01. FLS, fibroblast-like synoviocytes; OA, osteoarthritis; RA, rheumatoid arthritis; SEM, standard error of the mean; siNC, control siRNA.

Since previous studies have shown that IL-8 is induced by either IL-1β or TNF-α in RA FLS [19-21], we asked whether IL-1β or TNF-α contributes to IL-8 production stimulated by Cyr61 in FLS. We first found that the levels of IL-1β and TNF-α were remarkably higher in RA SF (Figure 3A), which is consistent with our previous reports [29,38]. Next, we assessed the role of IL-1β and TNF-α in IL-8 production in FLS induced by Cyr61. We employed a specific siRNA to knockdown IL-1β or TNF-α expression in FLS and examined IL-8 production induced by Cyr61. The results showed that, compared with control siRNA treatment (siNC), IL-8 mRNA expression was not reduced in IL-1β or TNF-α-knockdown FLS when exogenous Cyr61 was added (Figure 3B and 3C). These results suggest that IL-1β and TNF-α are not likely to take part in the process of IL-8 production from Cyr61-induced FLS; in other words, Cyr61-promoted IL-8 production in RA FLS in an IL-1β and TNF-α independent pathway.

### Cyr61-induced IL-8 secretion by FLS stimulated the migration of neutrophils

**Figure 4:**
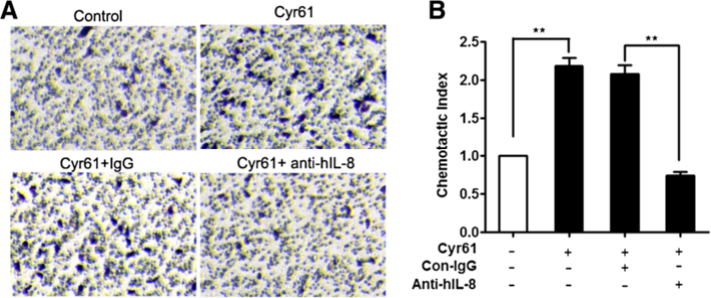
**The culture supernatant from Cyr61-treated FLS promotes neutrophil migration.** The FLS were treated with Cyr61 protein (5 μg/ml) for 48 hours and the culture supernatant was harvested. After that, the supernatant was pre-treated with anti-IL-8 antibody (5 μg/ml) or control IgG (5 μg/ml) for 1 hour and used for a chemotaxis assay as described in Methods. **(A)** Neutrophils that migrated in response to the culture supernatant from Cyr61-treated FLS were stained with crystal violet. **(B)** The culture supernatant from Cyr61-treated FLS promoted neutrophil migration and this chemotactic response was inhibited by anti-IL-8 antibody. The results are expressed as the chemotactic index, being the number of cells that migrated towards the sample divided by the number of cells that migrated towards the control medium. Data represent the mean ± SEM of at least three independent experiments. * P <0.05, ** P <0.01. FLS, fibroblast-like synoviocytes; SEM, standard error of the mean.

Next, we further explored whether Cyr61-induced IL-8 secretion by FLS has functional activity for inducing neutrophil migration. Using a chemotaxis assay, we found that the supernatant from Cyr61-treated FLS significantly augmented the chemotaxis of neutrophils isolated from peripheral blood of healthy individuals (Figure 4A and 4B). We further preincubated Cyr61-treated supernatant with specific IL-8 neutralizing (or control) Ab, and the results showed that addition of a neutralizing Ab against IL-8 in the supernatant indeed reduced the number of migrating neutrophils (Figure 4B), indicating that Cyr61-induced IL-8 secretion by FLS has neutrophil chemoattractant activity. This allowed us to show that Cyr61 induces IL-8 production which further leads to promoting neutrophil migration. Taken together, our data demonstrate that, in addition to IL-1β and TNF-α, Cyr61 is a newly identified inducer of IL-8 in RA FLS, suggesting that Cyr61 might be involved in the inflammatory and tissue damage induced by infiltrating neutrophils.

### Blocking Cyr61 ameliorated inflammation and down-regulated the expression of MIP-2 *in vivo*

**Figure 5:**
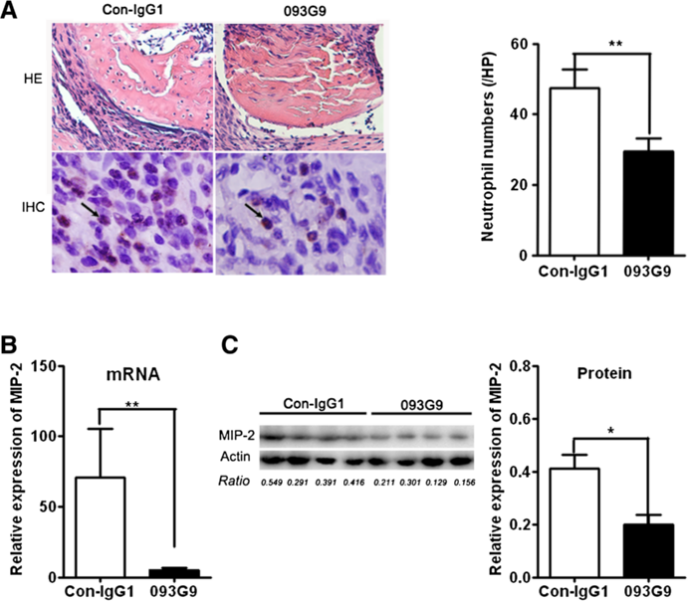
**Decreased neutrophil infiltration and MIP-2 production of CIA mice treated with anti-Cyr61 antibody. (A)** Immunohistochemistry of joint tissue from CIA mice treated with 093G9 or con-IgG1. Left panel: Representative photomicrographs showing H & E staining (Up, 100×) and infiltrating neutrophils stained with mouse neutrophil marker anti-Gr1 Ab (Lower, 400×) in joint tissue from CIA mice treated with con-IgG1 or 093G9. Arrow points to representative neutrophils (red). Right panel: The number of infiltrating neutrophils in joint tissue from CIA mice treated with con-IgG1 (open bar, n = 6) or 093G9 (black bar, n = 6) were counted in six randomly selected fields per tissue slice by optical microscope. **(B)** MIP-2 mRNA relative expression in synovial tissues from CIA mice treated with control IgG1 (open bar, n = 6) or 093G9 (black bar, n = 6) was evaluated by real-time PCR. **(C)** Left panel: MIP-2 protein level in synovial tissues from CIA mice treated with control IgG1 (n = 4) or 093G9 (n = 4) was detected by western blotting. Right panel: The density of each band was quantified by densitometric scanner and the relative amount of MIP-2 protein was calculated from the ratio of each band value to β-actin. Data represent the mean ± SEM from three independent experiments. * P <0.05, ** P <0.01. CIA, collagen-induced arthritis; SEM, standard error of the mean.

As we found that Cyr61-stimulated FLS promoted IL-8 expression *in vitro*, we asked whether Cyr61 indeed plays a role in IL-8 expression and relevant inflammation *in vivo.* We established a CIA mice model and treated them with 093G9 (Cyr61 neutralization Ab) [39]. Our previous reports revealed that the inflammatory score was significantly decreased and leukocyte infiltration and synovial hyperplasia in joints were ameliorated in 093G9-treated CIA mice [29]; similar results were obtained in this study and are shown in Figure 5A and Additional file 1: Figure S3. Moreover, microscopy showed that infiltrating neutrophils were obviously decreased in the mice treated with 093G9 (Figure 5A). Next, we analyzed the expression of MIP-2 (a counterpart of human IL-8) in joints of the mice with CIA and found that blocking Cyr61 also down-regulated the expression of MIP-2 (Figure 5B and 5C). This suggests that blocking Cyr61 activity reduced neutrophil migration into target tissues of CIA mice by down-regulating MIP-2 activity, resulting in the amelioration of joint inflammation and erosion.

### Cyr61-induced IL-8 production in FLS depends on AKT, JNK and ERK1/2 activation

**Figure 6:**
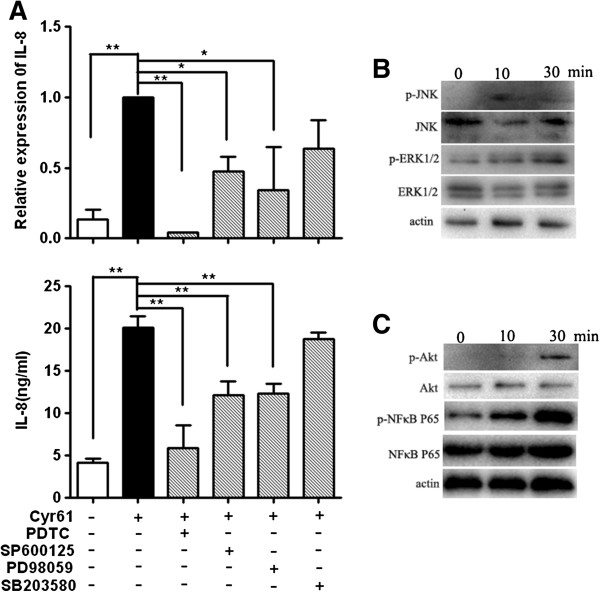
**Signaling pathways involved in Cyr61 regulating IL-8 production in RA FLS. (A)** Effect of inhibitors of signaling pathways on Cyr61-induced IL-8 expression. Up: FLS were treated with 4 μM PDTC, 20 μM SP600125, 1 μM PD98059 or 10 μM SB203580 in combination with Cyr61 (5 μg/ml) (shadow bars) for 2 hours, and IL-8 mRNA relative expression was evaluated by real-time PCR. Down: RA FLS were treated with 4 μM PDTC, 20 μM SP600125, 1 μM PD98059 or 10 μM SB203580 in combination with Cyr61 (5 μg/ml) (shadow bars) for 48 hours, and IL-8 protein level was examined by ELISA. Control (open bar), Cyr61 (no inhibitors, black bar). Data represent the mean ± SEM of at least three independent experiments. * P <0.05, ** P <0.01*.***(B)** Phosphorylation of JNK and ERK1/2 was detected by western blotting. Lane 1: unstimulated FLS, lane 2 and lane 3: stimulated with Cyr61 (5 μg/ml) for 10 minutes and 30 minutes, respectively. **(C)** Phosphorylation of AKT and NF-κB was detected by western blotting. Lane 1: unstimulated FLS, lane 2 and lane 3: stimulated with Cyr61 (5 μg/ml) for 10 minutes and 30 minutes, respectively.

Since the results showed that Cyr61 directly induced IL-8 production in FLS, we probed the downstream signaling pathway(s) using known inhibitors of several pathways, including PDTC (inhibitor of NF-κB activation), SP600125 (inhibitor of JNK), PD98059 (inhibitor of ERK1/2) and SB203580 (inhibitor of p38 MAPK). The results showed that Cyr61-stimulated IL-8 mRNA and protein expression in FLS were markedly decreased in the presence of the JNK, ERK1/2 and NF-κB inhibitors. In contrast, inhibition of p38 MAPK activities showed no effect on Cyr61-induced IL-8 production (Figure 6A). Further analysis showed that Cyr61 treatment led to a dramatic increase in the phosphorylation level of the JNK, ERK1/2 and NF-κB p65 subunit in FLS (Figure 6B and 6C) and enhanced NF-κB nuclear translocation as shown by laser scanning confocal immunofluorescence microcopy (Additional file 1: Figure S4). Previous studies in breast cancer cells suggested that Cyr61 could induce NF-κB activation via the PI3K/AKT pathway [23,40,41]. We, thus, checked whether this pathway was also activated in FLS upon Cyr61 stimulation. Indeed, we found that the phosphorylated (activated) form of AKT was strongly enhanced in response to Cyr61 treatment in FLS (Figure 6C). Based on these results, we suggest that Cyr61 induced IL-8 production in FLS depends on AKT, NF-κB, JNK and ERK1/2 signaling pathways.

### Cyr61 increased c-Jun, C/EBPβ and p65 binding to the response element in the IL-8 promoter

**Figure 7:**
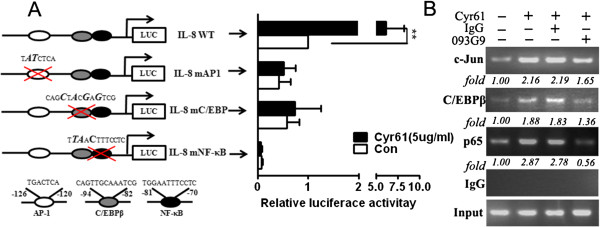
**Identification of binding sites essential for transcription of IL-8 in Cyr61 treated FLS. (A)** Wild-type (IL-8WT) and mutant vectors (mAP-1, mC/EBPβ and mNF-κB) were cotransfected with the control vector pRL-TK into HSF cells for 4 hours, cultured for 20 hours and incubated for 2 hours in the presence or absence of Cyr61 (5 μg/ml). The luciferase activity relative to control was indicated after normalization with Renilla luciferase activity. Data represent the mean ± SEM of at least three independent experiments. * P <0.05, ** P <0.01*.***(B)** FLS were stimulated with Cyr61 (5 μg/ml), and the c-Jun, C/EBPβ and p65 bound to the response element in the IL-8 promoter were detected by ChIP assay. It is noted that pre-incubation of cells with 093G9 (20 μg/ml) or control IgG (20 μg/ml) 1 hour before the Cyr61 stimulation suppressed the c-Jun, C/EBPβ and p65 DNA binding. The relative amount of IL-8 promoter DNA bound to c-Jun, C/EBPβ and p65 was detected by PCR and quantified by densitometric scanner. ChIP, chromatin immunoprecipitation; FLS, fibroblast-like synoviocytes; HSF, human skin fibroblasts; IgG, immunoglobulin G; SEM, standard error of the mean.

Studies have shown that IL-8 expression is regulated by a sequence spanning nucleotides -1 to -133 of the upstream DNA flanking the IL-8 gene, and that this region contains response elements for AP-1, C/EBP and NF-κB and is essential and sufficient for IL-8 expression [37,42]. To further identify the molecular mechanism responsible for Cyr61 induced IL-8 expression, we first constructed an IL-8 promoter (IL-8WT, which includes response elements for AP-1, C/EBP and NF-κB) with upstream of the luciferase gene. Further we transfected IL-8WT promoter into human skin fibroblasts (HSFs) and then treated the HSFs with Cyr61. The results showed that Cyr61 increased the IL-8WT promoter activity about seven-fold (Figure 7A), suggesting that Cyr61 is able to activate IL-8 promoter and enhance IL-8 gene expression. To further analyze the role of three known transcription factors (AP-1, C/EBP, and NF-κB) in the control of IL-8 gene expression in response to Cyr61 stimulation, we transfected the IL-8 promoter with mutations (IL-8mAP-1, IL-8mC/EBP and IL-8mNF-κB, which inhibit the binding site for AP-1, C/EBP and NF-κB, respectively) into HSFs, then treated HSFs with 5 μg/ml Cyr61 for two hours. The results showed that, upon stimulation with Cyr61, luciferase activity was not significantly increased compared with untreated HSFs (Figure 7A). These data indicate that AP-1, C/EBP and NF-κB binding motifs are essential for the Cyr61-induced IL-8 gene expression in RA FLS. To detect the *in vitro* binding of c-Jun, C/EBPβ and p65 to the IL-8 promoter following Cyr61 challenge, we performed a ChIP assay and examined the binding of c-Jun, C/EBPβ and p65 to the IL-8 promoter in FLS stimulated with exogenous Cyr61. The results showed that the levels of transcription factors (c-Jun, C/EBPβ and p65) binding to the IL-8 promoter in FLS were increased significantly compared with controls (Figure 7B). In contrast, treatment with 093G9 resulted in reducing binding of c-Jun, C/EBPβ and p65 to the IL-8 promoter region significantly (Figure 7B). Together, these results indicate that Cyr61 induced c-Jun, C/EBPβ and p65 binding to the corresponding response elements in the IL-8 promoter and increased the transcriptional activity of the IL-8 promoter. Based on these findings, we suggest that Cyr61 induced IL-8 production in FLS via AKT, JNK and ERK1/2 dependent AP-1, C/EBP and NF- κ B activation.

## Discussion

Although Th17 cells are newly identified inflammation cells in the pathogenesis of RA [43-45], a number of studies have revealed that neutrophils also play a pivotal role in the initiation and progression of RA [2-5]. As the most abundant cells infiltrating either in the SF of the affected joints or at the pannus/cartilage interface in RA, neutrophils are able to release cytotoxic mediators, cytokines and chemokines into the site of inflammation, leading to tissue damage and cartilage destruction [2-5,36]. Moreover, recently, some studies showed that neutrophils have an interaction with Th17 cells [46] and can release IL-17 in inflamed ST [11,12], adding a novel role for neutrophils in the initiation of RA. Considering that promoting neutrophil migration into the site of inflammation is critical for strengthening the cross-talk between neutrophils and Th17 cells, finding new inducers for increasing production of IL-8, a strong chemoattractant for neutrophil recruitment, is critical for developing a new strategy for RA treatment.

CCN1/Cyr61, as a member of the growth factor-inducible immediate-early genes, belongs to the CCN family [22,27] and is known as a novel pro-inflammatory factor [47-50]. Our studies have established that over-expressed Cyr61 not only stimulates FLS proliferation in an autocrine manner [28], but also initiates Th17 cell differentiation by promoting IL-6 production in FLS [29]. Considering that FLS are a source of Cyr61 and other inflammatory proteins [51], we asked whether Cyr61 is involved in IL-8 production by FLS in RA.

In this study, we first examined the amount of neutrophils infiltrated in SF and ST derived from RA patients. The results suggested that neutrophils were abundant in both SF and ST, which is consistent with previous reports [36]. Further, we found that Cyr61 was able to induce IL-8 mRNA expression and increase protein synthesis in FLS from RA patients. Given that studies have shown that IL-1β or/and TNF-α induce IL-8 production in RA FLS [19-21], we evaluated whether IL-1β and TNF-α were involved in the Cyr61-induced IL-8 production in FLS. By RNAi technology, we demonstrated that Cyr61-promoted IL-8 production was not dependent on an IL-1β and TNF-α pathway. As IL-8 is a critical chemokine that functions in promoting neutrophil migration, we tested whether Cyr61-induced IL-8 in RA FLS could stimulate neutrophil migration and found that it was indeed the case. Taken together, these results strongly indicate that Cyr61 induces IL-8 production by an IL-1β and TNF-α independent pathway, promotes the migration of neutrophils into joints and enhances the initiation and progression of RA inflammation. Indeed, in our study, we found that administration of a specific anti-Cyr61 antibody in CIA mice not only ameliorated inflammation, but also down-regulated the expression of MIP-2 (a counterpart of human IL-8) and impaired the infiltration of neutrophils in ST *in vivo*.

It is known that IL-1β and TNF-α are very important cytokines in inflammation and tissue damage that promote the synthesis of inflammatory proteins, including IL-8 for recruiting neutrophils [20,52]. Anti-IL-1β and TNF-α treatments in RA show efficacy in inhibiting inflammation and tissue erosion [53,54]. Nevertheless, some side-effects of cytokine-based therapy have been reported, including susceptibility to serious infection and malignancies [53,54]. Thus, it is very essential to find new therapeutic options for the treatment of RA. Our current study revealed that Cyr61, as extracellular matrix produced by FLS, promotes IL-8 in an IL-1β and TNF-α independent manner; blocking Cyr61 action might be of benefit by avoiding the side-effects of anti-IL-1/TNFα-based therapy. Together with our previous findings that Cyr61 promotes FLS proliferation and IL-6 production, Cyr61 plays a critical role in the inflammation and tissue damage caused by RA, suggesting that targeting Cyr61 may be an effective means for the treatment of RA.

**Figure 8:**
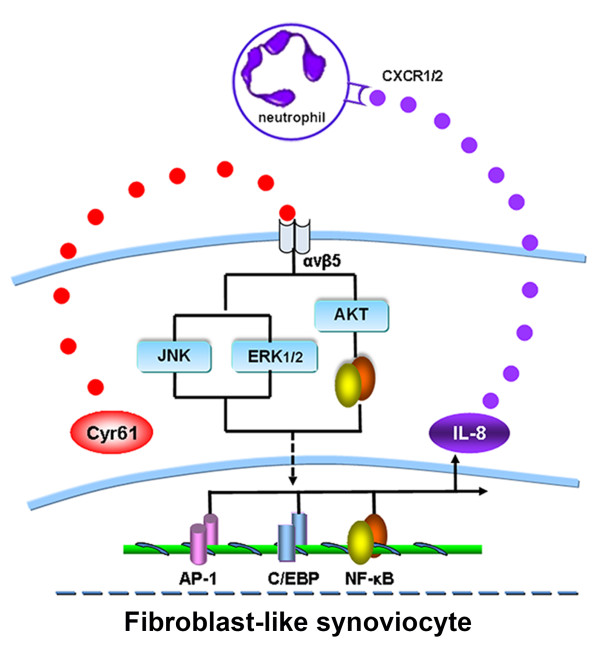
**A schematic model for Cyr61-stimulated IL-8 production and its role in the migration of neutrophils.** Cyr61 secreted by FLS stimulates IL-8 production via AKT, JNK and ERK1/2-dependent AP-1, C/EBP and NF-κB signaling pathways. Production of IL-8 recruits an abundance of neutrophils to the site of inflammation. In turn, neutrophils are able to release cytotoxic mediators, cytokines and chemokines into the diseased joints, leading to tissue damage and cartilage destruction. Thus, Cyr61 might act as a novel proinflammatory factor contributing to the inflammation of RA via stimulation of neutrophil migration. FLS, fibroblast-like synoviocytes; RA, rheumatoid arthritis.

According to the present and previous studies, Cyr61 produced by RA FLS can initiate a novel cross-talk among FLS, neutrophils and Th17 cells, whereby Cyr61 acts to stimulate IL-8 production by FLS, and the increased IL-8 then acts to promote the migration of neutrophils (Figure 8). Importantly, a recent study showed that there is an interaction between human neutrophils and Th17 cells in inflammatory diseases [46]. This new cross-talk, together with the regulation of Cyr61 production in FLS by IL-17 and Th17 cell differentiation by Cyr61-induced IL-6 in FLS that we had previously reported [28,29], forms a new positive feedback loop and vicious cycle in promoting neutrophil accumulation, Th17 cell differentiation and FLS proliferation. Cyr61 plays key roles in this vicious cycle; in other words, Cyr61 plays important roles in RA pathogenesis.

### How does Cyr61 induce IL-8 production in FLS?

Activation of MAPK and NF-κB pathways have been shown to contribute to IL-8 expression [37,42,55,56], but the role of MAPK and the NF-κB pathway for the Cyr61-induced IL-8 production in FLS remains to be determined. To address the signaling pathway of Cyr61 promoting IL-8 production in FLS, we evaluated the profile of AKT/NF-κB, a well-known Cyr61/integrins pathway [57], and three well-defined MAPK pathways (JNK, ERK and p38). As expected, AKT/NF-κB pathways contributed to Cyr61-induced IL-8 production in FLS. However, the analysis of MAPK pathways indicated that JNK and ERK pathways were involved in the Cyr61-induced IL-8 production in FLS. Interestingly, the p38 pathway was not found to contribute to the Cyr61-induced IL-8 production in FLS. Previous observations suggested that, in the IL-1β or TNF-α induced IL-8 production, the p38 MAPK pathway contributes to IL-8 gene expression by stabilizing mRNAs in RA FLS [42,58]. Our study first shows that the p38 MAPK pathway was not involved in the Cyr61-induced IL-8 production in FLS; in other words, signaling cascades of Cyr61-induced IL-8 production are different from signaling pathways of IL-1β or TNF-α-induced IL-8 production. Considering the role of the p38 MAPK pathway in post transcriptional regulation of IL-8 production, how to stabilize the mRNAs of IL-8 in Cyr61-stimulated FLS is under investigation.

Based on the results of Cyr61-induced IL-8 production in FLS via JNK, ERK and NF-κB activation, we examined the transcriptional mechanisms regulated by Cyr61. Although it is well known that the core IL-8 promoter contains binding sites for AP-1, C/EBP and NF-κB, the different binding activity of AP-1, C/EBP and NF-κB on the IL-8 promoter has been attributed to different IL-8 production in the cells [37,42]. We performed promoter-reporters and ChIP analysis for testing regulatory elements of the IL-8 promoter in Cyr61-treated FLS. The results showed that AP-1/c-Jun, C/EBPβ and NF-κB binding to the IL-8 promoter were all necessary for Cyr61-induced IL-8 expression in RA FLS. Earlier studies have documented that transcription factors involved in IL-8 gene transcription interact to facilitate the formation of an enhanceosome-like structure that favors the induction of the IL-8 promoter [42]. In our studies, we found that Cyr61 enhanced AP-1, C/EBPβ and NF-κB binding to the IL-8 promoter simultaneously, suggesting that signaling pathways mediated by Cyr61 provoke an interaction among these transcription factors and may contribute to the formation of an enhanceosome-like structure for IL-8 production in RA FLS, even though the p38 MAPK pathway was not active in Cyr61-induced IL-8 production in RA FLS. Based on these results, we propose that Cyr61 is able to induce IL-8 production similar to pro-inflammatory cytokines, by which Cyr61 enhances neutrophil infiltration in joints with RA.

Although a recent study showed that hypoxia might induce Cyr61 and IL-8 secretion in nasal polyp fibroblasts, no direct evidence demonstrated that Cyr61 induces IL-8 production under an inflammatory environment via an IL-1β/TNF-α independent pathway [59]. Considering that Cyr61 expression may be up-regulated as a protective response to hypoxia *in vivo*[60], it would be interesting to investigate whether hypoxia can enhance Cyr61-induced IL-8 production by RA FLS.

## Conclusions

Our study indicated for the first time, that Cyr61 is a novel IL-8 production inducer and initiates the pathogenesis mediated by neutrophils. Combining the observation that infiltrating neutrophils and Th17 form an inflammatory cross-talk with our previous findings that Cyr61 promotes Th17 development and FLS proliferation [28,29,46], we suggest that Cyr61 plays a key role in the vicious cycle formed by interaction among activated Th17, proliferated FLS and infiltrating neutrophils in the development of RA. Thus, targeting Cyr61 might be an effective strategy in RA treatment.

## Abbreviations

Ab: Antibody; bp: base pair; ChIP: Chromatin immunoprecipitation; CIA: Collagen-induced arthritis; (D)MEM: (Dulbecco’s) modified Eagle’s medium; ECM: Extracellular matrix; ELISA: Enzyme-linked immunosorbent assay; FLS: Fibroblast-like synoviocytes; H & E: Hematoxylin and eosin; HRP: Horseradish peroxidase; HSFs: Human skin fibroblasts; Ig: Immunoglobulin; IL: Interleukin; mAb: Monoclonal antibody; MAPK: Mitogen-activated protein kinase; OA: Osteoarthritis; PBS: Phosphate-buffered saline; PDTC: Pyrrolidine dithiocarbamate; RA: Rheumatoid arthritis; RT-PCR: Real-time polymerase chain reaction; SF: Synovial fluid; SiNC: Control siRNA; siRNA: Small interfering RNA; ST: Synovial tissues; TNF-α: Tumor necrosis factor-α.

## Competing interests

The authors declare that they have no competing interests.

## Authors’ contributions

Study conception and design were performed by XZ, LX, RH and NL; acquisition of data was done by JZ, JL, JX, STS, YH, YS, ZZ and BS, and analysis and interpretation of data, by XZ, LX and NL. NL had full access to all of the data in the study and takes responsibility for the integrity of the data and the accuracy of the data analysis. All authors were involved in drafting the manuscript or revising it critically for important intellectual content. All authors read and approved the final manuscript.

## Supplementary Material

Additional file 1 **A PDF file containing all supplementary tables and figures as referred to in the main text.** The additional file contains supplementary **Table S1.** (‘Clinical characteristics of RA and OA patients’), **Table S2.** (‘Specific primers used in real-time PCR analysis’), **Table S3.** (‘Sequences of siRNA used in RNAi knockdown of gene expression’) and **Table S4.** (‘The primers used for construction of luciferase reporter plasmids’), and supplementary **Figure S1.** (‘The occurrence and distribution of infiltrating cells in synovial tissue of RA patients’), **Figure S2.** (‘The sequence for siCyr61 was evaluated’), **Figure S3** (‘Ameliorated severity of CIA mice treated with anti-Cyr61 antibody’) and **Figure S4.** (‘Cyr61 induced nuclear translocation of NF-κB in FLS’). Click here for file
