# Supplementary material for: Cyr61 is involved in neutrophil infiltration in joints by inducing IL-8 production by fibroblast-like synoviocytes in rheumatoid arthritis
Source: Arthritis Res Ther. 2013 Nov 13;15(6):R187. doi: 10.1186/ar4377 (PMC3978874; doi:10.1186/ar4377)
Supplement: Additional file 1 — A PDF file containing all supplementary tables and figures as referred to in the main text. The additional file contains supplementary Table S1. (‘Clinical characteristics of RA and OA patients’), Table S2. (‘Specific primers used in real-time PCR analysis’), Table S3. (‘Sequences of siRNA used in RNAi knockdown of gene expression’) and Table S4. (‘The primers used for construction of luciferase reporter plasmids’), and supplementary Figure S1. (‘The occurrence and distribution of infiltrating cells in synovial tissue of RA patients’), Figure S2. (‘The sequence for siCyr61 was evaluated’), Figure S3 (‘Ameliorated severity of CIA mice treated with anti-Cyr61 antibody’) and Figure S4. (‘Cyr61 induced nuclear translocation of NF-κB in FLS’). [file ar4377-S1.pdf]

**SUPPLEMENTARY TABLES**

**Table S1. Clinical characteristic of RA and OA patients**

| *Clinical data* | *RA* | *OA* |
|---|---|---|
| Sex | | |
| Male | 6 | 11 |
| Female | 40 | 16 |
| Age (years) | 57.1±12.1 | 71.1±7.2 |
| Disease duration (years) | 16.4±9.2 | 9.0±7.1 |
| Tender joints [a] | 13.4±5.6 | 1.29±0.4 |
| Swollen joints [a] | 14.2±5.2 | 1.46±0.6 |
| Physical function scores [b] | 0.63±0.28 | / |
| Patients assessment [c] | 7.1±1.4 | / |
| Physician assessment [c] | 6.4±1.2 | / |
| HSS score [d] | 45.3±9.6 | 53.4±9.2 |
| RF (IU/ml) | 160.7±131.4 | / |
| CRP (μg/ml) | 23.9±19.9 | 9.1±12.7 |
| ESR (mm/h) | 47.7±31.5 | 24.7±39.4 |

[a] Twenty-eight joints were assessed for tenderness, and twenty-eight were assessed for swelling. [b] The modified Standard Health Assessment questionnaire was used. Scores can change from 0 to 3, with higher scores indicating greater disease activity. [c] A 0–10 visual-analogue scale was used in which higher values indicated more severe abnormalities.[d] The Hospital for Special Surgery Knee Score (HSS) was used. Scores can change from 0 to 100, with lower scores indicating greater disease activity. Values represent the mean ± SD.

**Table S2. Specific primers used in real-time PCR analysis**

| *Name* | *Primer* | *Sequence(5'-3')* |
|---|---|---|
| GAPDH | FW | CACATGGCCTCCAAGGAGTAA |
| | RV | TGAGGGTCTCTCTCTTCCTCTTGT |
| IL-8 | FW | CCAAGGAAAA CTGGGTGCAGAG |
| | RV | GGCACAGGGGAACAAGGACTTG |
| IL-1β | FW | TTGTTGAGCCAGGCCTCTCT |
| | RV | ACCAAATGTGGCCGTGGTT |
| TNF-α | FW | TGGCCCAGGCAGTCAGA |
| | RV | GGTTTGCTACAACATGGGCTACA |
| Cyr61 | FW | TCCAGCCCAACTGTAAACATCA |
| | RV | GGACACAGAGGAATGCAGCC |

**Table S3. Sequences of siRNA used in RNAi knockdown of gene expression**

| *siRNA* | | *Sequence(5'-3')* |
|---|---|---|
| Cyr61 | sense | CAACGAGGACUGCAGCAAATT |
| | antisense | UUUGCUGCAGUCCUCGUUGAG |
| IL-1β | sense | GGUGAUGUCUGGUCCAUAUTT |
| | antisense | AUAUGGACCAGACAUCACCTT |
| TNF-α | sense | GCCUGUAGCCCAUGUUGUATT |
| | antisense | UACAACAUGGGCUACAGGCTT |

**Table S4. The primers used for construction of luciferase reporter plasmids**

| *Name* | *Primer* | *Sequence(5'-3')* |
|---|---|---|
| IL-8 (mAP-1) | FW | GTGAGATCTGAAGTGTGAT**AT**CTCAGG |
| | RV | GTGAAGCTTGAAGCTTGTGTGCTCTGC |
| IL-8 (mC/EBP) | FW | GTGAGATCTGAAGTGTGATGACTCAGGTTTGC |
| | | CCTGAGGGGATGGGCCATCAG**C**T**A**C**G**A**G**TCG |
| | RV | GTGAAGCTTGAAGCTTGTGTGCTCTGC |
| IL-8 (mNF-KB) | FW | GTGAGATCTGAAGTGTGATGACTCAGGTTTGC |
| | | CCTGAGGGGATGGGCCATCAGTTGCAAATCG |
| | | T **TA**A**C**TTTCCTCT |
| | RV | GTGAAGCTTGAAGCTTGTGTGCTCTGC |

The bold and underlined regions correspond to the mutation of target IL-8 promoter for IL-8 (mAP-1), IL-8 (mNF-κB) and IL-8 (mC/EBP).

**SUPPLEMENTARY FIGURES**

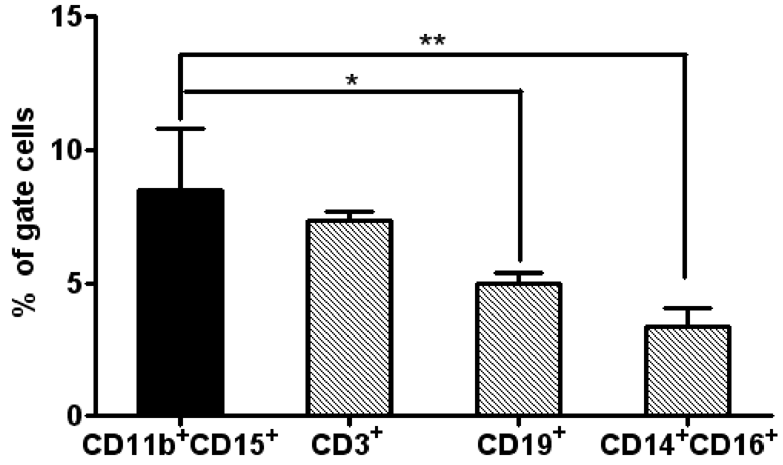


**Figure S1 The occurrence and distribution of infiltrating cells in synovial tissue of RA patients.** The percentage of infiltrating cells in synovial tissue from RA patients was detected by FCS. Isolation and staining of cells from ST as described in Materials and Methods. Data are representative of at least 3 independent experiments. ** P<0.05, ** P<0.01*

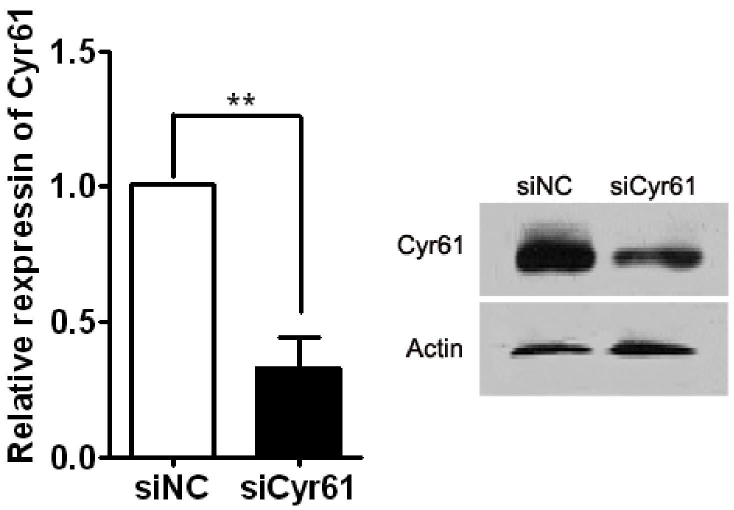


**Figure S2. The sequence for siCyr61 (small interfering RNA against Cyr61) was evaluated.** Expression of Cyr61 mRNA (Left panel) and the Cyr61 protein (Right panel) was inhibited in FLS treated with specific siCyr61. FLS treated with scrambled siRNA (siNC) were used as controls. Data are representative of at least 3 independent experiments. ** *P<0.01*

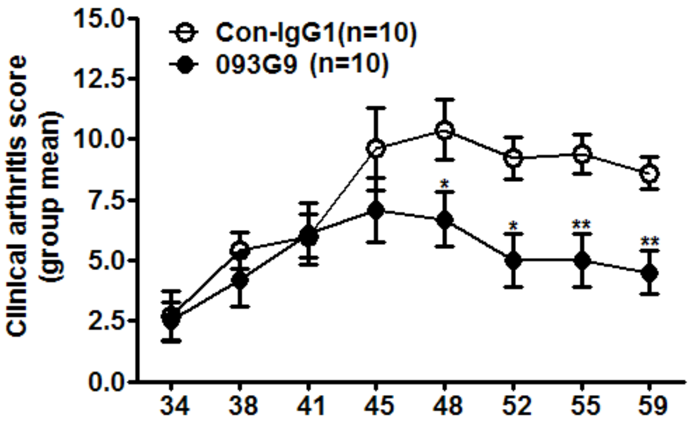


**Figure S3 Ameliorated severity of CIA mice treated with anti-Cyr61 antibody.**
CIA mice were injected with either con-IgG1 or anti-Cyr61 mAb 093G9, twice a week starting at day 34 and continuing until day 59, and clinical score and severity of CIA were monitored and scored as described in Materials and Methods. Anti-Cyr61 mAb 093G9 significantly decreased arthritis severity compared with that in IgG1-treated mice. Control (○), 093G9 (●). Data represent the mean ± SEM of 10 mice per group. * *P<0.05*, ** *P<0.01*

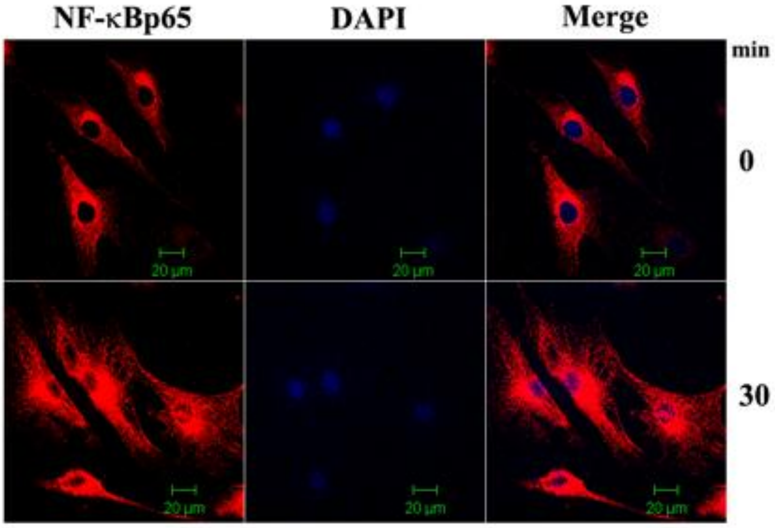


**Figure S4 Cyr61 induced nuclear translocation of NF-κB in FLS.** Up: unstimulated FLS, down: stimulated with Cyr61 (5 μg/ml) for 30 min. NF-κB was detected by anti-p65 (red). Nuclei were stained with DAPI (blue). Merged picture shows NF-κB translocation into the nucleus. The data represent one of the three independent experiments.
